# Small extracellular vesicles-transported lncRNA TDRKH-AS1 derived from AOPPs-treated trophoblasts initiates endothelial cells pyroptosis through PDIA4/DDIT4 axis in preeclampsia

**DOI:** 10.1186/s12967-023-04346-6

**Published:** 2023-07-24

**Authors:** Qian Chen, Jiexing He, Haihua Liu, Qiuyu Huang, Shuoshi Wang, Ailan Yin, Shuying Chen, Xinyang Shen, Yanxuan Xiao, Haoyue Hu, Jiayi Jiang, Wenqian Chen, Song Wang, Zhenqin Huang, Jiaqi Li, You Peng, Xiaocong Wang, Xinping Yang, Zhijian Wang, Mei Zhong

**Affiliations:** 1 Department of Obstetrics & Gynecology, Nanfang Hospital, Southern Medical University, Guangzhou, 510515 China; 2 Key Laboratory of Mental Health of the Ministry of Education, Southern Medical University, Guangzhou, 510515 China; 3 Department of Bioinformatics, School of Basic Medical Sciences, Southern Medical University, Guangzhou, 510515 China; 4 Department of Obstetrics, Shenzhen People’s Hospital, (The Second Clinical Medical College, Jinan University, The First Affiliated Hospital, Southern University of Science and Technology), Shenzhen, 518020 China; 5 Department of Obstetrics, Shenzhen Second People’s Hospital, Shenzhen University 1st Affiliated Hospital, Shenzhen, 518035 China

**Keywords:** Preeclampsia, Advanced oxidation protein products, Extracellular vesicles, lncRNA, TDRKH-AS1, PDIA4, DDIT4, Oxidative stress, Pyroptosis

## Abstract

**Background:**

Substantial studies have demonstrated that oxidative stress placenta and endothelial injury are considered to inextricably critical events in the pathogenesis of preeclampsia (PE). Systemic inflammatory response and endothelial dysfunction are induced by the circulating factors released from oxidative stress placentae. As a novel biomarker of oxidative stress, advanced oxidation protein products (AOPPs) levels are strongly correlated with PE characteristics. Nevertheless, the molecular mechanism underlying the effect of factors is still largely unknown.

**Methods:**

With the exponential knowledge on the importance of placenta-derived extracellular vesicles (pEVs), we carried out lncRNA transcriptome profiling on small EVs (sEVs) secreted from AOPPs-treated trophoblast cells and identified upregulated lncRNA TDRKH-AS1 as a potentially causative factor for PE. We isolated and characterized sEVs from plasma and trophoblast cells by transmission electron microscopy (TEM), nanoparticle tracking analysis (NTA) and western blotting. The expression and correlation of lncRNA TDRKH-AS1 were evaluated using qRT-PCR in plasmatic sEVs and placentae from patients. Pregnant mice injected with TDRKH-AS1-riched trophoblast sEVs was performed to detect the TDRKH-AS1 function in vivo. To investigate the potential effect of sEVs-derived TDRKH-AS1 on endothelial function in vitro, transcriptome sequencing, scanning electron Microscopy (SEM), immunofluorescence, ELISA and western blotting were conducted in HUVECs. RNA pulldown, mass spectrometry, RNA immunoprecipitation (RIP), chromatin isolation by RNA purification (ChIRP) and coimmunoprecipitation (Co-IP) were used to reveal the latent mechanism of TDRKH-AS1 on endothelial injury.

**Results:**

The expression level of TDRKH-AS1 was significantly increased in plasmatic sEVs and placentae from patients, and elevated TDRKH-AS1 in plasmatic sEVs was positively correlated with clinical severity of the patients. Moreover, pregnant mice injected with TDRKH-AS1-riched trophoblast sEVs exhibited a hallmark feature of PE with increased blood pressure and systemic inflammatory responses. Pyroptosis, an inflammatory form of programmed cell death, is involved in the development of PE. Indeed, our in vitro study indicated that sEVs-derived TDRKH-AS1 secreted from AOPPs-induced trophoblast elevated DDIT4 expression levels to trigger inflammatory response of pyroptosis in endothelial cells through interacting with PDIA4.

**Conclusions:**

Herein, results in the present study supported that TDRKH-AS1 in sEVs isolated from oxidative stress trophoblast may be implicated in the pathogenesis of PE via inducing pyroptosis and aggravating endothelial dysfunction.

**Supplementary Information:**

The online version contains supplementary material available at 10.1186/s12967-023-04346-6.

## Background

Preeclampsia (PE) is a life-threatening pregnancy complication, recognized by new-onset hypertension and organ symptoms after 20 weeks’ gestation, like cardiovascular, hepatorenal, coagulation and neurological systems dysfunction, with an incidence of 2% to 4% worldwide [1]. Maternal and perinatal morbidity and mortality attributable to adverse pregnancy are increasing with PE being a pivotal contributor [2]. To date, there is no definitive treatment besides the termination of pregnancy [1]. Herein, investigation of the pathogenesis and development mechanisms of PE is extremely crucial to maternal and fetal health.

A rich body of literatures elucidates that the two-stage process can decipher the pathogenesis of PE. The shallow implantation of trophoblast and impaired spiral artery remodeling elicit placental hypoxia and stress, which not only causes release of circulating factors but also affects placenta development [3, 4]. Endothelia dysfunction and systemic inflammatory are thought to be trigged by the circulating factors resulting in clinical syndrome characteristic of PE [5, 6]. Nevertheless, the molecular mechanisms underlying the effect of circulating factors remain to be further explored. As evidenced by the increasing number of publications on placental extracellular vesicles (pEVs) which are present in the maternal circulation detected as early as at 6 weeks of gestation, they have been built to link the pathogenesis of pregnancy disorders, especially PE [7]. EVs are categorized as microvesicles (MVs), exosomes (Exos) and apoptotic bodies (Abs), based on their size and biogenic pathway [8]. In addition, MISEV (minimum information for the study of extracellular vesicles) guideline have recommended that physical characteristics, such as size, are used for classify EVs without biogenic synthesis evidence[9]. In general, EVs have defined as small EVs (sEVs) with size < 220 nm and medium/large EVs (m/lEVs) with size ≥ 220 nm [7]. Mounting studies have revealed that pEVs markedly elevated in PE patient are engaged in endothelial dysfunction, imbalanced angiogenesis and systemic inflammation, all of which eventually aggravate the disease development [10–14]. Notably, pEVs with various bioactive cargos such as proteins, nucleic acids and lipids can impair endothelial function resulting in systemic inflammation response in PE [15, 16].

Increasing numbers of studies have focused on contents of pEVs involved in the pathogenesis of PE. The lipid characteristics of pEVs are found and play an essential role in PE via mediating macrophage inflammatory polarization [17]. Additionally, pEVs carry endothelial nitric oxide synthase (eNOS) that can contribute to lower nitric oxide (NO) activity inducing endothelial dysfunction in PE [18]. Strikingly, EVs derived lncRNAs are known to participate in various pathological processes of diseases, including PE [19, 20]. Trophoblast exosomal lncRNA UCA1 can recruit USP14 to PFN1 via RhoA/Rho-kinase (ROCK) pathway, resulting in endothelial injury of PE [16]. Herein, we conducted lncRNAs sequencing of sEVs extracted from trophoblasts under oxidative stress conditions to further decipher the pathogenesis of PE. LncRNA TDRKH-AS1 is one of the lncRNAs we have identified as upregulated in sEVs derived from oxidative stress-induced trophoblast, which is involved in cancer cell invasion and proliferation [21].

As we know, increased release of pEVs stimulated by trophoblast stress especially oxidative stress are detected in PE women causing maternal systemic manifestations [7]. Placental oxidative stress is well documented to play a pivotal role in the pathophysiology of PE [22]. Advanced oxidation protein products (AOPPs) as promising biomarkers of oxidative stress-induced damage have been detected abundantly in various chronic inflammatory diseases [23, 24]. Encouragingly, our previous studies have demonstrated that the expression of AOPPs were significantly elevated in placenta and plasma from PE women which was correlated with PE severity. Moreover, we found that AOPPs induced trophoblast dysfunction involving in the development of PE [25, 26]. However, whether AOPPs-induced placenta damage impairs vascular endothelial cells resulting in systemic inflammation of PE has not been illuminated.

Substantial studies have revealed that placenta under oxidative stress of PE patient can active NOD-like receptor protein 3 (NLRP3) inflammasomes which recruit apoptosis-associated speck-like protein containing a caspase recruitment domain (ASC) and caspase-1 [27, 28]. Gasdermin D (GSDMD)-NT cleaved by caspase-1 induces cell swelling and lysis accompanied by cell membrane rupture and cellular contents release such as enzyme lactate dehydrogenase (LDH). Upon inflammatory caspase, a plethora of caspase-1 active form promotes pro-interleukin-1β (IL-1β) and pro-IL-18 to mature forms, leading to massive inflammatory processes and consequently resulting in pyrotosis [29]. As a programmed cell death, pyroptosis is involved in multiple cardiovascular diseases accompanied by vascular endothelial cells injury [30]. However, whether pyroptosis impairs endothelial cell function and thus contributes to systemic inflammation and manifestation of PE are rarely investigated.

Here, we report our study on the role of sEVs-derived TDRKH-AS1 isolated from AOPPs-induced trophoblast on endothelial function in vivo and invitro. We found that trophoblast sEVs-derived TDRKH-AS1 elicited endothelial cells pyroptosis via PDIA4/DDIT4 inducing systemic inflammatory response, involved in processing of PE. Our study might shed promising light on the pathogenesis of this disease with the hope of improving early screening and treatment.

## Methods

### Plasma and placenta collection from preeclampsia patients and normal controls

This work has been approved by The Research Ethics Board of Nanfang Hospital, Southern Medical University, China (NFEC-2020-155) and all patients have signed informed consent. The 60 plasma and placental samples were collected from December 2020 to April 2022 at Department of Obstetrics & Gynecology of Nanfang Hospital. The clinical characteristics of PE patient were strictly followed the International Society for the Study of Hypertension in Pregnancy (ISSHP). The peripheral blood was collected in an EDTA tube and centrifuged at 2000*g* for 20 min at 4 °C to obtain plasma. The collection and processing of the placental tissue were conducted following our previous study [31]. The plasma and placentae were stored at − 80 °C for later experiments.

### Cell culture and AOPPs-induced oxidative stress cell model

HTR8/SVneo cell line and HUVECs were obtained from American Type Culture Collection (Manassas, USA). HTR8/SVneo cell line in RPMI 1640 medium (Corning, USA) and HUVECs in DMEM (Gibco, USA) were cultured supplemented with 10% fetal bovine serum (Gibco, USA) in humidified air at 37 °C with 5% CO_2_.

In our previous studies, detailed AOPPs preparation have been described, and evident cells damage were triggered in HTR8/SVneo cells with AOPPs (200 μg/ml) for 48 h [25, 26]. Hence, this concentration and time were used to incubate HTR8/SVneo cells for building oxidative stress cells model. The cell culture supernatants of HTR8/SVneo cells cultured with EVs-free FBS were collected to isolate sEVs in subsequent experiments. HUVECs were incubated with sEVs (100 μg/mL) derived from trophoblasts for 48 h. The supernatants and cells were harvested for further use.

### Extracellular vesicles isolation and characterization

Plasma sEVs were extracted using ExoQuick exosome precipitation kit (EXOQ5TM, System Biosciences, USA) following the manufacturer's protocol as presented in our previous study [32]. The suspensions of cells were sequentially centrifuged at 300*g* for 10 min, 2000*g* for 10 min and 10,000*g* for 30 min at 4 °C to remove cells and cellular debris. The supernatants were filtered with the 0.22 μm filters, followed by centrifuging at 100,000*g* for 1 h. The pellets were washed using PBS and centrifuged at 100,000*g* for 1 h. The sEVs pellets from plasma and cell culture medium were resuspended with PBS and stored at − 80 °C for further experiments. sEVs characterization was performed using nanoparticle tracking analysis (NTA), transmission electron microscopy (TEM) and western blotting analysis as our previous work [32].

### sEVs transcriptome analysis and enrichment analysis

RNA sequencing was performed at RIBOBIO (Guangzhou, China) using Illumina HiSeq 2500. We first removed reads with adapters, reads with > 10% of unknown bases and low-quality reads (sequencing quality < 10). After filtering, the remaining clean reads were subjected to the bioinformatics analysis. The clean reads were first mapped to the Homo sapiens rRNA database using the bowtie2 alignment software, to remove the remaining rRNA reads. The non-rRNA reads were used to perform the transcriptome assembling and quantification. We downloaded reference genome (GRCh38) and gene model annotation files from GENCODE database (https://www.gencodegenes.org/human/). The reads from the clean data of RNA-seq were aligned to the human reference genome (GRCh38) with STAR. The HTseq-count was used to quantify gene expression profile. Raw counts of genes with > 10 counts among samples were used for downstream analysis. Differential expression analysis was performed by DESeq2 and edgeR, and a cutoff of *p* < 0.05 was used to determine the differential expression genes (DEGs). KEGG pathway enrichment analysis and GO (Gene Ontology) enrichment analysis were performed by clusterProfiler.

### RNA isolation and quantitative real-time PCR

Total RNA was isolated using TRIzol (Invitrogen, USA). The reverse transcription and qRT-PCR were conducted with the HiScript Reverse Transcription Kit and ChamQ SYBR Green qPCR Kit (Vazyme, China) in a LightCycler 480 (Roche, Swiss) system to quantify genes expression according to the manufacturer’s instruction. The specific primers sequences were listed in Additional file 9: Table S8. The relative gene expression was calculated using 2^−△△CT^ method and converted to fold changes using ACTB or U1 as internal controls.

### Lentiviral expression constructs and transfection

The full length and shRNAs targeting of lncRNA TDRKH-AS1 and PDIA4 were synthesized and cloned into pGC-FU vector (Genechem, China). All the lentiviral vectors were purchased from Genechem company (Genechem, China). The full length of lncRNA TDRKH-AS1 was synthesized and cloned into the pGC-FU vector/GV502 at the restriction sites AgeI/BamHI, with the vector elements (polyA-MCS-UBI) RV-SV40-EGFP-IRES-puromycin. The coding sequence (CDS) of PDIA4 was synthesized and cloned into the pGC-FU vector/GV350 at restriction sites BamHI/AgeI with the vector elements Ubi-MCS-3FLAG-SV40-Neomycin. The interference sequence (GCAGATCAAGAAACAGAAC) of lncRNA TDRKH-AS1 was designed and cloned into the pGC-FU vector/GV493 at restriction sites AgeI/EcoRI, with the vector elements hU6-MCS-CBh-gcGFP-IRES-puromycin. The interference sequence (GCTTGTGTTGACCAAAGAGAA) of PDIA4 was designed and cloned into the pGC-FU vector/GV152 at restriction sites AgeI/EcoRI, with the vector elements hU6-MCS-CMV-Neomycin. HTR8/SVneo cells and HUVECs were transfected these plasmids according to the manufacturer’s protocols, followed by selecting using puromycin or G418 (Gibco, USA).

### Animal experiments

C57BL/6 J female and male mice (8 weeks) were purchased from SiPeiFu Biotechnology (China) maintained in the environment of constant temperature with 12 h light/dark cycle and free access to water/chow. This project was conducted following animal protocol procedures approved by the Department of Laboratory Animal Sciences, Southern Medical University (L2020101), and the animals were handled in accordance with the guiding principles published in the National Institutes of Health Guide for the Care of Animals and the Institutional Animal Care and Use Committee.

After adaptive feeding for 1 week, the C57BL/6 J female and male mice were pair housed in a ratio of 2:1 overnight. Vaginal plug formation was first determined as embryonic day 0.5 (E0.5 d). The pregnant mice were randomly divided into eight groups of six each and injected with NG-nitro-L-arginine methyl ester(L-NAME) (125 mg/kg/100uL), AOPPs (50 mg/kg/100uL), PBS (100uL), sEVs (100 μg/100uL) derived from AOPPs-induced trophoblasts, control trophoblasts, overexpression TDRKH-AS1 trophoblasts and overexpression control trophoblasts. Control mice received no injections. The sEVs-treated dams were administered daily from E11.5 d to E15.5 d via the tail vein. Simultaneously, the L-NAME-treated mice by subcutaneous injection and AOPPs/ PBS-treated groups through a tail vein were performed daily from E7.5 d to E17.5 d. Systolic blood pressure (SBP) of conscious mice under stable condition was determined by tail cuff plethysmography using the Softron BP 2010 (Softron Biotechnology, China) on nine time point (pre-pregnancy, E0.5, E4.5, E7.5, E9.5, E11.5, E13.5, E15.5 and E17.5). The placenta, fetus and plasma of each group were harvest on E17.5 d for further experiments. The weight and appearance of uterine horn, fetus and placenta were recorded.

### PKH26-labelled sEVs uptake

Trophoblasts sEVs were stained with PKH26 (Sigma-Aldrich, USA) following the manufacturer’s instructions and incubated with HUVECs for 24 h at 37 °C. The cells were subsequently fixed with 4% paraformaldehyde and washed using PBS. After being immersed with 0.5% Triton-X 100, HUVECs were stained in FITC Phalloidin (Solarbio, China) and DAPI (Solarbio, China). The uptake of PKH26-labelled sEVs in HUVECs was detected with a confocal laser scanning microscopy (Olympus, Japan). For the mice uptake experiments, the pregnant mice were injected with PKH26-labelled sEVs or PBS for 24 h via tail vein. After anesthetizing the mice, bioluminescence imaging results of mice, uterine horn, fetus and placenta were conducted to visualize the sEVs uptake using Bruker In-Vivo Imaging Systems and analyzed with Bruker MI SE software (Bruker Corp, USA).

### Hematoxylin and Eosin

Placental tissues were fixed in 4% paraformaldehyde, and then treated with dimethylbenzene and alcohol, followed by stained with hematoxylin and eosin. Results were obtained at 200X under a microscope (Olympus, Tokyo, Japan).

### Enzyme-linked immunosorbent assay (ELISA) and lactate dehydrogenase activity assay

The concentrations of IL-1β and IL-18 in cell culture supernatants or plasma were detected using the ELISA kits (MEIMIAN, China), and the plasma contents of AOPPs were assessed via the ELISA kit (MLBIO, China). The processes were conducted according to the manufacturer's instructions. The LDH activity in the supernatants of HUVECs was detected using LDH activity detection kit (Jiancheng Bioengineering Institute, China) following the manufacturer’s protocol. The absorbance OD value was measured by the microplate reader (SpectraMax I3x, USA).

### Overexpression TDRKH-AS1 transcriptome and enrichment analysis

RNA sequencing was performed at Novogene (Beijing, China) using Illumina NovaSeq. The reads from the clean data of RNA-seq were aligned to the human reference genome (GRCh38) with STAR. The HTseq-count was used to quantify gene expression profile. Raw counts of genes with > 10 counts among samples were used for downstream analysis. Differential expression analysis was performed by DESeq2 and edgeR, and a cutoff of adjusted *p* < 0.05 was used to determine the differential expression genes (DEGs). KEGG pathway enrichment analysis and GO (Gene Ontology) enrichment analysis were performed by clusterProfiler.

### Genes collection and analysis

Inflammatory genes were collected from Gene Ontology (GO) and MSigDB database, using the key words “inflammatory” or inflammation. Pyroptosis associated genes were collected from GO, MSigDB and genecards databases, using keywords “pyroptosis”. All enrichment analyses were performed on the R platform, and one-tailed Fisher’s exact test was used. Error bars represent the standard deviation of the fraction, estimated with a bootstrapping method with 100 re-samplings.

### Scanning electron microscopy (SEM)

The cells were fixed with 2.5% glutaraldehyde, rinsed in 0.1 M PBS for 45 min and postfixed in the dark for 2 h using 1%OsO_4_ at room temperature. After being dehydrated and dried, the specimens were mounted on stubes and sputter-coated with gold–palladium. The images were monitored by SEM (ZEISS, Germany).

### Immunofluorescence

The cells were fixed in 4% paraformaldehyde for 20 min and permeabilized with 0.3% Triton X-100 for 15 min. After being washed with PBS, the cells were blocked with 5% BSA for 1 h and incubated with the primary antibodies against NLRP3 (1:200, 19771-1-AP, Proteintech, China) and GSDMD (1:100, 20770-1-AP, Proteintech, China) at 4 °C overnight. The cells were washed with PBS, and then incubated with fluorochrome-conjugated secondary antibody (1:400, Proteintech, China) for 1 h in the dark. Finally, the cells were stained with DAPI (Solarbio, China) and analyzed using a confocal laser scanning microscopy (Olympus, Japan).

### RNA pulldown and mass spectrometry data analysis

TDRKH-AS1 and its antisense RNA were made from transcription with the Biotin RNA Labeling Mix (Roche, USA) and T7 RNA polymerase (Roche, USA). Biotinylated RNA was incubated with HUVECs nuclear extracts, and pulldown proteins were run on SDS-PAGE gels (Sigma, USA) and stained with silver staining solution (Beyotime, China) as our previous work [31]. The lncRNA pulled-down complex was sent to the company (Wininnovate Bio, China), using mass spectrometry to detect proteins interacting with *TDRKH-AS1*. We determined the proteins interacting with *TDRKH-AS1*, satisfying the criteria: only exists in the sense probe pulled-down complex and with at least 3 peptides. KEGG pathway enrichment analysis and GO (Gene Ontology) enrichment analysis were performed by clusterProfiler.

### RIP assays

RNA pulldown experiments were carried out using EZ-Magna RIP Kit (Millipore, USA) according to manufacturer’s instruction. HUVECs were collected by centrifugation, and the cell pellets were then lysed in RIP lysis buffer. Anti-PDIA4 antibodies and normal lgG (Millipore, USA) were used for immunoprecipitation, and the immunoprecipitated RNA was analyzed by qRT-PCR as previously described [31]. The sequences of two specific primers were listed in Additional file 9: Table S8.

### Bioinformatics prediction

PDIA4 is located on reverse strand, the transcription start site was chr7:149,028,505. The region, ranged from chr7:149028005 (downstream 500 bp) to chr7:149029505 (upstream − 1000 bp), was used in the prediction of the TDRKH-AS1 binding sites. LongTarget was used to predict TDRKH-AS1 DNA-binding motifs (triplex-forming oligonucleotides, TFOs) and binding sites (triplex target sites, TTSs) [33]. The binding regions of best TFO (TFO1) were used to downstream experiments. The sequences of TFO1 and binding sites of PDIA4’s promoter are listed in Additional file 10: Table S9.

### Chromatin isolation by RNA purification (ChIRP)

ChIRP assay was conducted using a Magna ChIRP Chromatin Isolation by RNA Purification Kit (Sigma-Aldrich, USA) according to the manufacturer’s instructions. Briefly, cell lysate was ultrasonicated. After centrifugation, the supernatant was reacted with probes and complete hybridization buffer for 4 h at 37 ℃. The cocktail mixed with streptavidin magnetic beads (Sigma-Aldrich, USA) was washed using wash buffer for five times. Then qPCR and qRT-PCR were used to detect the interaction between lncRNA TDRKH-AS1 and PDIA4 promoter with purified DNA and RNA. We have designed primers for region1 (chr7:149028308-149028504) and region2 (chr7:149028576-149028711), which covered four binding sites (closed to the transcriptional start site). The probes and primers are listed in Additional file 10: Table S9.

### Plasmid and siRNA transfection

The plasmids pcDNA3.1-DDIT4 and si-DDIT4 were obtained from Genechem (Genechem, China). The transfection was conducted by Lipofectamine 3000 (Invitrogen) following manufacturer’s instruction. HUVECs transfected for 48 h were harvested to subsequent studies. The siRNAs sequences were shown in Additional file 9: Table S8.

### Western blot assays

Total proteins were extracted using RIPA lysis buffer with PMSF, phosphatase and protease inhibitors. Protein concentration was determined by BCA Protein Assay Kit (KeyGEN, China). The extracted proteins were separated to SDS-PAGE (8–10%) and electro-blotted on the polyvinylidene difluoride membrane. After blocked with 5% skim milk, the membranes were incubated with the primary antibodies against CD63 (1:1000, ab134045, Abcam, USA), CD9 (1:1000, ab236630, Abcam, USA), TSG101 (1:1000, ab125011, Abcam, USA), PLAP (1:10,000, ab133602, Abcam, USA), Calnexin (1:1000, ab133615, Abcam, USA), NLRP3 (1:1000, #15101, Cell Signaling Technology, USA), Cleaved Caspase-1 (1:1000, #4199, Cell Signaling Technology, USA), Cleaved GSDMD(1:1000, #36425, Cell Signaling Technology, USA), ASC (1:5000, 10500-1-AP, Proteintech, China), PDIA4 (1:1000, NBP2-90208, NOVUS, USA), DDIT4 (1:1000, ab191871, Abcam, USA) and β-Actin (1:1000, #4970, Cell Signaling Technology, USA) at 4 °C overnight. After washed with TBST, the membranes were incubated with secondary antibody (1:2000, Proteintech, China) for 1 h. Target proteins were detected using ECL kit (Millipore, USA) by an imaging system (Biolight, China).

### Coimmunoprecipitation (Co-IP)

The cells lysis was obtained by the coimmunoprecipitation kit (Thermo Scientific, USA). After being centrifuged at 14,000*g* for 15 min, the supernatants were incubated with indicated antibodies at 4 °C overnight, and then incubated with the protein A/G-agarose beads at 4 °C for 2 h. The mixtures were centrifuged at 3000*g* for 2 min and washed using washing buffer for three times, followed by western blotting analysis. Anti-PDIA4 (NBP2-90208, NOVUS, USA), anti-DDIT4 (ab191871, Abcam, USA) and anti-lgG (ab200699, Abcam, USA) were used as the primary antibodies.

### Quantification and statistical analysis

SPSS20.0 (IBM) was used to analysis all statistical results in this present work, and the data were presented as the mean ± S.D of three independent experiments. The comparison between the two independent groups was performed using Mann–Whitney or test Student’s t-test. Correlation analyses were conducted using Pearson correlation coefficient. *P* value less than 0.05 was considered statistically significant.

## Results

### Identification and characterization of sEVs isolated from AOPPs-induced trophoblast cells and maternal plasma

**Fig. 1:**
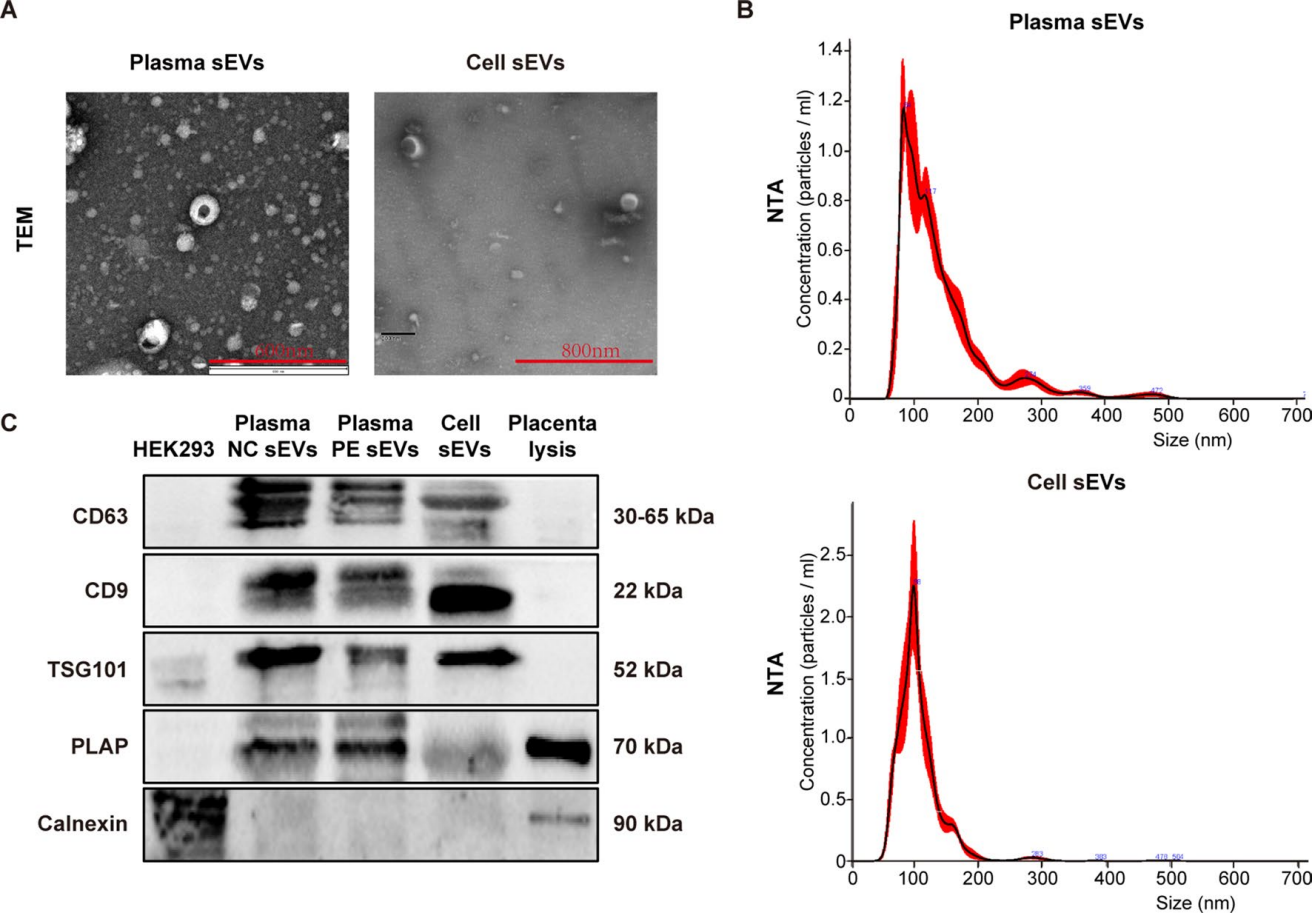
Characterization of isolated sEVs derived from trophoblast cells and maternal plasma. **A** Transmission electron microscopy analysis of sEVs morphology. Scale bar, 200 nm. **B** Nanoparticle tracking analysis of the sEVs size. **C** Western blotting analysis of the EV markers (CD63, CD9 and TSG101), PLAP and Calnexin expressions

Previously, we reported that AOPPs was highly expressed in the plasma and placenta of preeclampsia patients [25, 26]. To explore the molecular mechanisms underlying the AOPPs-induced injury in preeclampsia, HTR-8/SVneo cells were treated with AOPPs (200 μg/ml) for 48 h. Next, we isolated and purified sEVs from cell culture supernatants using standard ultracentrifugation for follow-up studies. We also collected 60 plasma and placental samples, 20 from EOSOE, 10 from LOSPE and 30 from normal controls. The clinical characteristics of the patients are summarized in Table 1 and Additional file 2: Table S1. Significantly elevated blood pressure and proteinuria were presented in the PE patients compared to the control group. The sEVs derived from plasma were isolated by exosome precipitation kit. To further validate our sEVs preparations, the isolated sEVs were characterized by TEM, NTA and western blotting. The cup-shaped and double-membrane structures of isolated particles were confirmed by TEM (Fig. 1A). The range of the sEVs size determined by the NTA was predominantly from 50 to 220 nm (Fig. 1B). The isolated sEVs were positive for the EV markers CD63, CD9 and TSG101, not Calnexin detected by the western blotting. Moreover, PLAP was expressed in these sEVs indicated that they were derived from placentae (Fig. 1C).

**Table 1:** Clinical characteristics of normal pregnancies and preeclamptic

Variable	Control(n = 30)	PE(n = 30)	*p* Value
Maternal age (year)	32.47 ± 4.622	31.97 ± 5.203	*p* = 0.695
Maternal weight (kg)	67.36 ± 7.987	72.07 ± 10.956	*p* = 0.062
Systolic blood pressure (mm Hg)	115.07 ± 8.153	171.27 ± 12.608	*p* < 0.01
Diastolic blood pressure (mm Hg)	73.57 ± 7.006	106.87 ± 11.673	*p* < 0.01
Proteinuria (g/day)	–	5.18 ± 4.393	–
Body weight of infant (g)	3255.0 ± 335.444	1966.3 ± 788.67	*p* < 0.01
Gestational age (weeks)	39.08 ± 0. 696	34.62 ± 3.378	*p* < 0.01
Placental weight (g)	573.33 ± 99.041	445.00 ± 98.313	*p* < 0.01
Fetal sex (Male), n (%)	53%	53%	
Fetal sex (Female), n (%)	47%	47%	
Primipara, n (%)	43%	27%	
Pluripara, n (%)	57%	73%

### The TDRKH-AS1 level is upregulated in sEVs derived from AOPPs-treated trophoblast cells and preeclamptic plasma

**Fig. 2:**
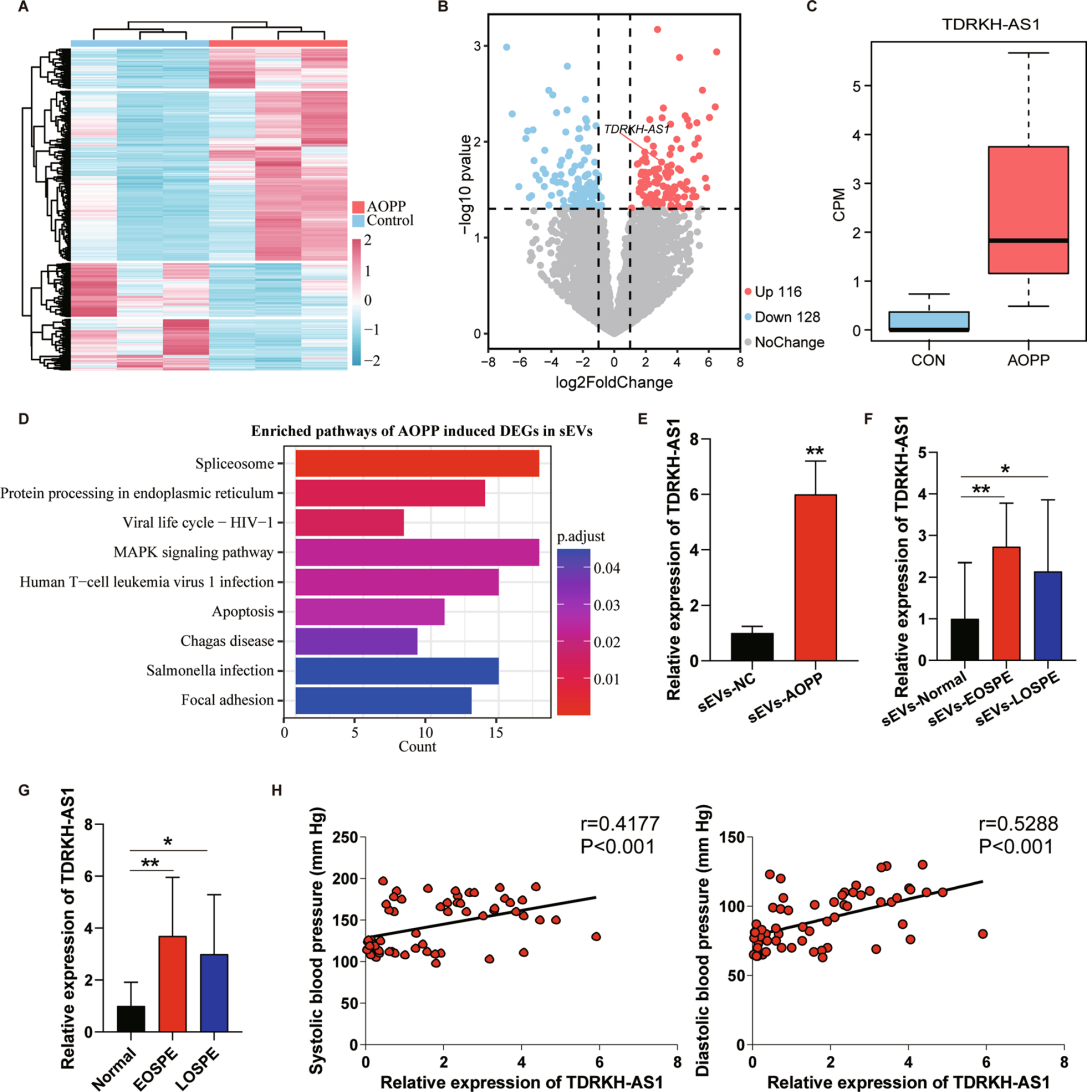
sEVs-derived TDRKH-AS1 as a potentially causative factors in PE. **A** The relative expression of DEGs across sEVs derived from AOPPs-treated trophoblast cells and control group. **B** The volcano plot shows the up-regulated lncRNAs (red) and down-regulated lncRNAs. **C** Boxplot of TDRKH-AS1 expression levels in sEVs sequencing. CPM: counts per million. **D** The enriched KEGG pathways of DEGs in sEVs sequencing. **E** The relative expression level of TDRKH-AS1 verified by qRT-PCR in sEVs secreted by AOPPs-treated trophoblast cells and control group. **F** The expression level of TDRKH-AS1 is up-regulated in plasmatic sEVs from EOSPE or LOSPE patients compared with normal group. **G** The TDRKH-AS1 expression levels in EOSPE or LOSPE placentae compared with normal placentae. **H** Positive correlation between systolic blood pressure and the relative expression of TDRKH-AS1 in plasmatic sEVs (r = 0.4177, *p* < 0.001). Positive correlation between diastolic blood pressure and the relative expression of TDRKH-AS1 in plasmatic sEVs (r = 0.5288, *p* < 0.001). The data are represented as the mean ± SD; **p < 0.01, *p < 0.05

We carried out high-throughput lncRNA/mRNA sequencing to detect the differential expression profile of sEVs-containing lncRNAs derived from AOPPs-treated trophoblast cells and control group, and found 976 differentially expressed genes (DEGs), including 651 up-regulated genes (116 lncRNAs) and 325 down-regulated genes (128 lncRNAs) (Fig. 2A, B). Most of differentially expressed lncRNAs (DElncRNA) were antisense and long intergenic non-coding RNAs (lincRNAs) biotypes (Additional file 3: Table S2). The lncRNA TDRKH-AS1 (10.4-fold increase) was among the top upregulated DElncRNAs in the sEVs derived from AOPPs-treated trophoblast cells (Fig. 2C). We further performed functional enrichment analysis for DEGs and found that these DEGs were involved in protein processing in endoplasmic reticulum, MAPK signaling pathway, apoptosis and focal adhesion (Fig. 2D, Additional file 4: Table S3). These pathways are reported to be extremely associated with preeclampsia, especially protein processing in endoplasmic reticulum [28]. The up-regulated expression of sEVs-derived TDRKH-AS1 was verified using qRT-PCR (six-fold increase) (Fig. 2E).

Next, we detected expression level of TDRKH-AS1 in collected plasmatic sEVs from patients and verified that both EOSPE patients and LOSPE patients showed significantly increased expression level (approximately 2.7-fold and 2.1-fold) of sEVs-derived TDRKH-AS1 (Fig. 2F). The about three~ fourfold increased expression level of TDRKH-AS1 further was also detected in EOSPE or LOSPE placentae compared to normal placentae using qRT-PCR (Fig. 2G). The plasmatic sEVs-containing TDRKH-AS1 expression level was positively correlated with the systolic and diastolic blood pressure of patients (Fig. 2H). Together, these results showed that sEVs-derived TDRKH-AS1 may be involved in the pathogenesis of preeclampsia.

### sEVs-transported TDRKH-AS1 induces preeclampsia-like features in pregnant mice

**Fig. 3:**
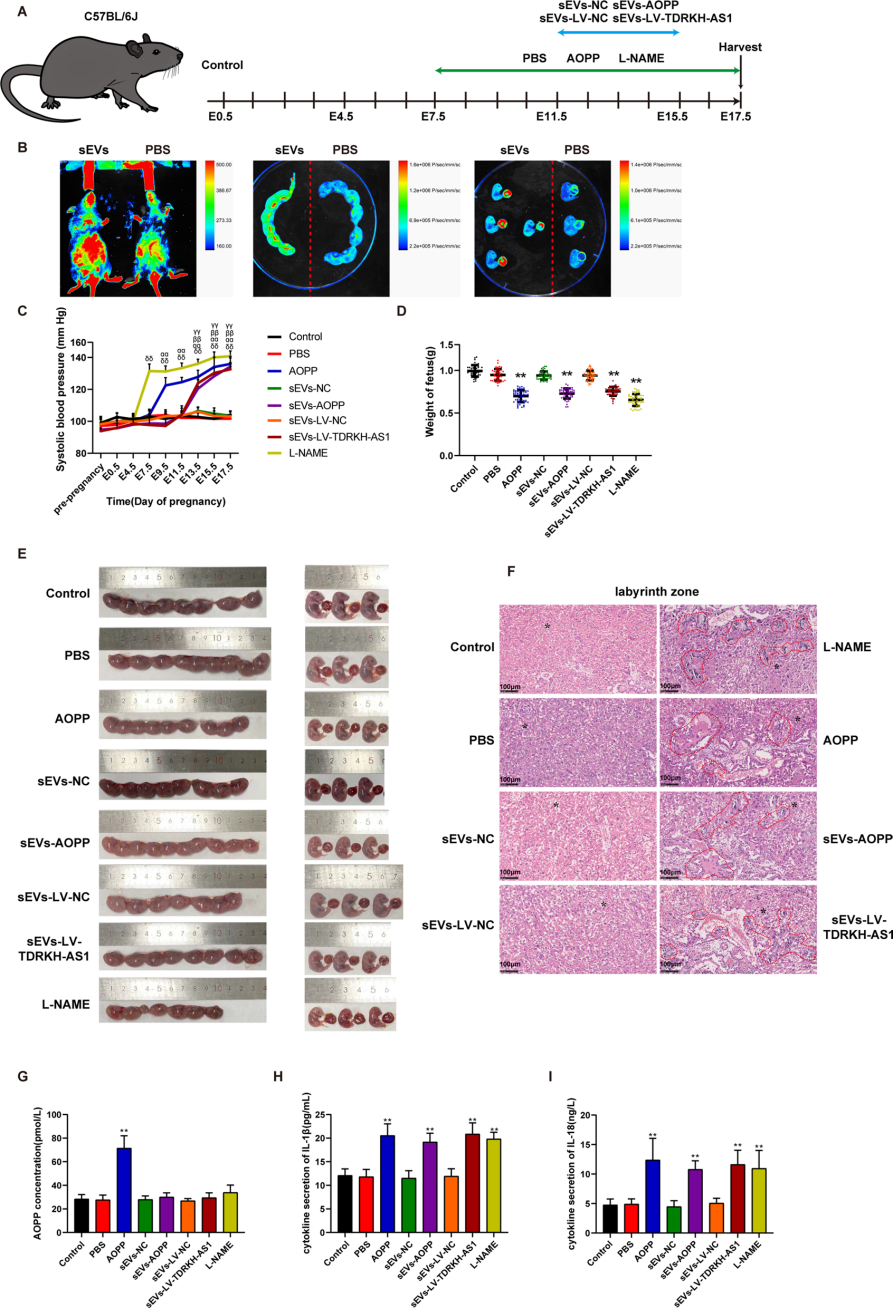
sEVs-derived TDRKH-AS1 induces preeclampsia-like symptoms in mice. **A** Schematic overview of the eight experimental groups. **B** The PKH26 red fluorescence using live animal imaging system. **C** The systolic blood pressure of the eight groups during pregnancy (n = 6, δ shows L-NAME treated mice vs. control, α shows AOPPs treated mice vs. PBS, β shows AOPPs-induced sEVs treated mice vs. sEVs control, γ shows LV-TDRKH-AS1 sEVs treated mice vs. sEVs LV-control, with.^δδ, αα, ββ, γγ^*p* < 0.01). **D** The fetal weights of L-NAME, AOPPs, LV-TDRKH-AS1 sEVs and AOPPs-induced sEVs treated groups are significantly declined compared with those of the corresponding control groups. **E** Representative images of uterine horn, fetus and placenta in each group. **F** Representative images of labyrinth zone of placenta using H&E-staining. Dashed lines and asterisk indicate typical structural change (original magnification, × 200; scale bar = 100 µm). **G**–**I** The concentration of AOPPs, IL-1β and IL-18 are quantified in maternal plasma of each group by ELISA assay. The data are represented as the mean ± SD; **p < 0.01

To further investigate the role of sEVs derived from AOPPs-treated trophoblast cells in vivo, pregnant mice were randomly divided into eight groups with six each injected with LV-TDRKH-AS1 sEVs, LV-NC sEVs, AOPPs-induced sEVs, NC sEVs, AOPPs, L-NAME, or PBS (Fig. 3A). We performed qRT-PCR to detect the relative expression level of TDRKH-AS1 in sEVs isolated from AOPPs-treated or established TDRKH-AS1 overexpression HTR8/SVneo cells (Additional file 1: Fig. S1A, B). As expected, upregulation of TDRKH-AS1 (5.5-fold and 8.9-fold) was confirmed in sEVs derived from two groups (Additional file 1: Fig. S1B). The PKH26 red fluorescence under live animal imaging system demonstrated that sEVs were distributed across systemic circulation of mice including that in placentae, whereas no or weak fluorescence(non-specific) was detected in mice injected with PBS (Fig. 3B). Relative to control or PBS groups, L-NAME and AOPPs treated group had approximately 28% and 18% increase in the systolic blood pressure (SBP) at E7.5 d and E9.5 d. Furthermore, the SBP was significantly increased by ~ 24% and ~ 22% in LV-TDRKH-AS1 sEVs and AOPPs-induced sEVs-injected mice compared with sEVs control groups from E13.5 d to the end of gestation (Fig. 3C). At E17.5 d, the mice were dissected to detect the change of fetuses and placentae. The decrease (34%, 26%, 20% and 23%, respectively) in fetal weight was observed in L-NAME, AOPPs, LV-TDRKH-AS1 sEVs and AOPPs-induced sEVs treated groups at E17.5 d (Fig. 3D, E).

Moreover, there were infracted, necrotic areas and narrowed blood vesicles in the labyrinth layer of L-NAME, AOPPs, LV-TDRKH-AS1 sEVs and AOPPs-induced sEVs treated placenta using HE staining (Fig. 3F). In order to exclude the effect of AOPPs in mice injected with sEVs, the concentrations of AOPPs were measured in plasma of each group. Only AOPPs treated mice had 2.6-fold elevated concentrations of AOPPs, other groups were not significantly altered (Fig. 3G). Interestingly, we found statistically higher concentration (1.7-fold and 2.4-fold) of inflammation-associated cytokines such as IL-1β and IL-18 in plasma of L-NAME, AOPPs, LV-TDRKH-AS1 sEVs and AOPPs-induced sEVs treated groups using ELISA (Fig. 3H–I). These findings indicated that AOPPs, AOPPs-induced sEVs and TDRKH-AS1 sEVs in circulation of mice resulted in adverse pregnancy outcomes, similar as detected in PE patients.

### TDRKH-AS1 initiates programmed inflammatory cell death in human umbilical vein endothelial cells (HUVECs)

**Fig. 4:**
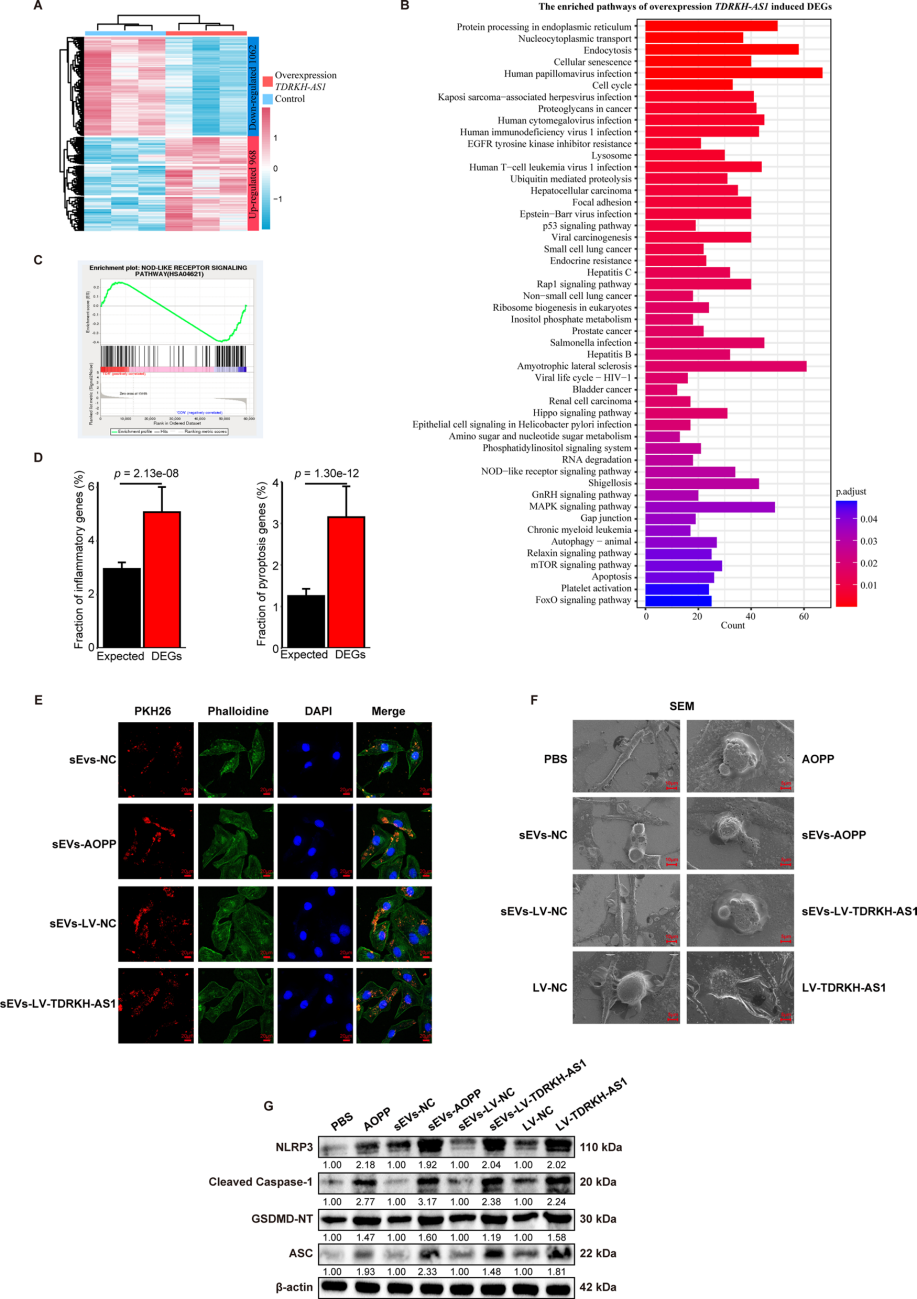
TDRKH-AS1 causes pyroptosis in HUVECs. **A** The relative expression pattern of DEGs induced by overexpression TDRKH-AS1 in HUVECs, including 968 up-regulated genes and 1062 down-regulated genes. **B** The enriched KEGG pathways of DEGs induced by overexpression TDRKH-AS1 in HUVECs. **C** Enrichment plots showing enrichment for NOD-like reception signaling pathway. **D** DEGs induced by overexpression TDRKH-AS1 are enriched with inflammatory/pyroptosis related genes. Error bars represent the standard deviation of the fraction, estimated using a bootstrapping method with 100 resampling. **E** The uptake of PKH26-labeled sEVs incorporates in HUVECs detected by confocal laser scanning microscopy. **F** The morphology of HUVECs is observed using scanning electronic microscopy. **G** The protein expression levels of NLRP3, cleaved-caspase-1, ASC and GSDMD-NT were measured by western blotting

In order to interrogate the functional impact of TDRKH-AS1 on HUVECs, we conducted transcriptome sequencing with overexpression of TDRKH-AS1 in HUVECs. The 2030 DEGs were found, including 968 up-regulated genes and 1062 down-regulated genes (Fig. 4A, Additional file 5: Table S4). Next, we performed functional KEGG pathway enrichment analyses, and found that these DEGs were enriched in protein processing in endoplasmic reticulum, cellular senescence, Rap1 signaling pathway and NOD − like receptor signaling pathway (Fig. 4B, Additional file 6: Table S5). There pathways are highly associated with inflammatory signaling [28, 34, 35]. Particularly, one of the enriched pathways is the NOD − like receptor signaling pathway, which regulates programmed inflammatory cell death, also pyroptosis (Fig. 4C). Moreover, 5.07% (103/2030) DEGs were inflammatory genes, showing significant enrichment compared with 2.96% (523/17698) in the expressed genes (*p* = 2.13e-08, One-tailed Fisher's exact test), and 4.15% (64/2030) DEGs were pyroptosis genes, showing significant enrichment compared with 1.25% (222/17698) in the expressed genes (*p* = 1.30e-12, One-tailed Fisher's exact test). These results suggest that TDRKH-AS1 may be involved in the gene regulation of inflammatory-related response, especially pyroptosis (Fig. 4D, Additional file 7: Table S6).

**Fig. 5:**
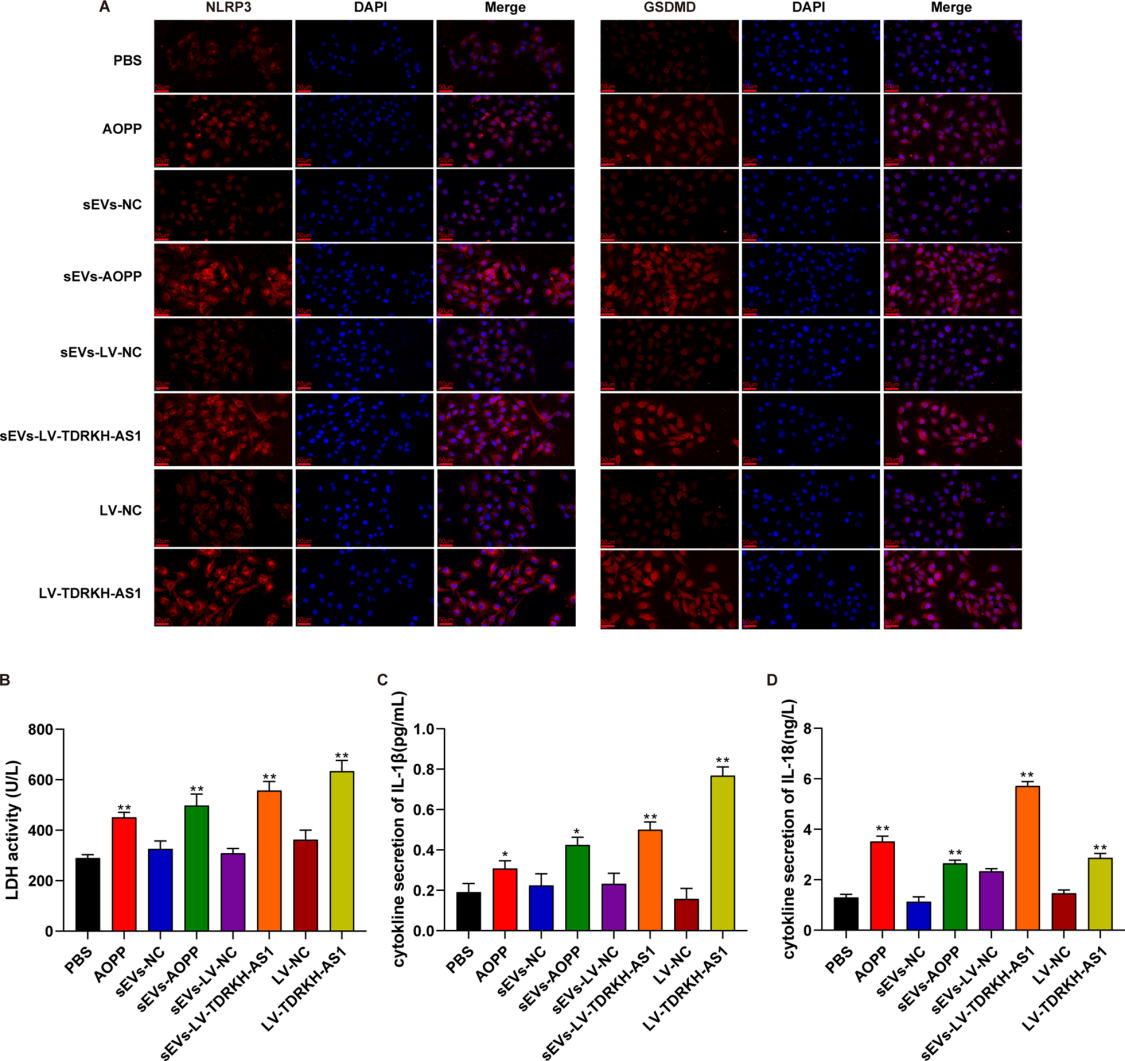
TDRKH-AS1 causes pyroptosis in HUVECs. **A** The detection of expression proteins is observed by confocal microscopy. NLRP3 and GSDMD antibody conjugated with Alexa 594, and nuclei was labeled with DAPI. (× 400; scale bar = 20 µm). **B** The levels of LDH release in each group. **C**, **D** The concentration of IL-1β and IL-18 in different treated HUVECs was detected by ELISA. The data are represented as the mean ± SD; **p < 0.01, *p < 0.05.

To further validate the above-described results, we performed a series of functional experiments. First, the PKH26-labeled sEVs incorporated in the cytoplasm of HUVECs after incubating for 24 h (Fig. 4E). Moreover, we detected the upregulated expression (4.7-fold and 8.3-fold) of TDRKH-AS1 in HUVECs after incubating with AOPPs-induced sEVs and LV-TDRKH-AS1 sEVs for 48 h (Additional file 1: Fig. S1B). To investigate functional effect of TDRKH-AS1 carried by sEVs on HUVECs, we established overexpression of TDRKH-AS1 and performed knockdown of this lncRNA using lentiviral transfection (Additional file 1: Fig. S1C) in HUVECs. SEM was used to observe the morphology of HUVECs treated with AOPPs, AOPPs-induced sEVs, LV-TDRKH-AS1 sEVs and overexpression TDRKH-AS1. The cells presented a pyroptosis-like morphology, with cell swelling, holes formation and membrane rupture (Fig. 4F). The detection of pyroptosis-associated proteins indicated that the expression levels of NLRP3, cleaved-caspase-1, ASC and GSDMD-NT were upregulated (2.1-fold, 2.3-fold, 1.8-fold and 1.9-fold) in the AOPPs, AOPPs-induced sEVs, LV-TDRKH-AS1 sEVs and overexpression TDRKH-AS1 groups compared with corresponding control groups using the western blotting (Fig. 4G). We further examined the similar expression of NLRP3 and GSDMD by immunofluorescence staining in each group (Fig. 5A). Moreover, the LDH release as an indicator of pyroptosis was quantified, in which the AOPPs, AOPPs-induced sEVs, LV-TDRKH-AS1 sEVs and overexpression TDRKH-AS1 groups significantly elevated ~ 66% of the LDH release levels (Fig. 5B). Additionally, the measurement of the pro-inflammatory cytokines IL-1β and IL-18 were significantly increased (2.6-fold and 2.4-fold) by treatment with AOPPs, AOPPs-induced sEVs, LV-TDRKH-AS1 sEVs and overexpression TDRKH-AS1 (Fig. 5C, D). These findings showed that TDRKH-AS1 derived from trophoblast sEVs under oxidative stress could induce pyroptosis in HUVECs.

### PDIA4 as a downstream effector of TDRKH-AS1

**Fig. 6:**
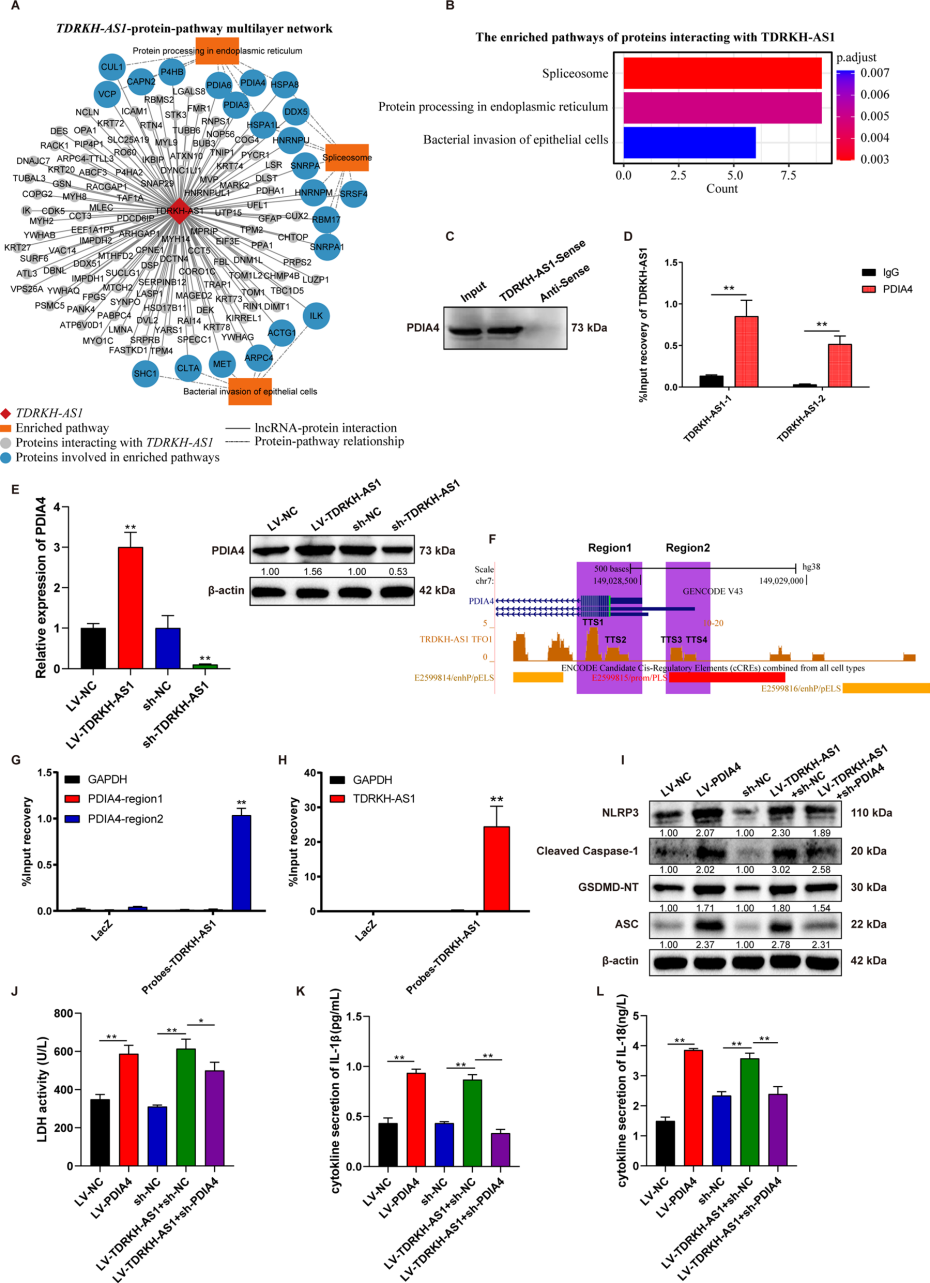
TDRKH-AS1 induces pyroptosis by binding to PDIA4 in HUVECs. **A** The TDRKH-AS1-protein-pathway multilayer interaction network, containing TDRKH-AS1, 138 proteins interacting with TDRKH-AS1 and 3 enriched pathway of proteins involved in, 138 lncRNA-protein interactions, and 24 protein-pathway relationships. **B** Enriched pathways of proteins interacting with TDRKH-AS1. **C** The interaction between TDRKH-AS1 and PDIA4 using RNA pull-down was validated by western blotting. **D** Binding proteins of TDRKH-AS1 detected by RIP assays and qRT-PCR. **E** TDRKH-AS1 overexpression increases the expression of PDIA4 on mRNA and protein levels, while TDRKH-AS1 knockdown showed the opposite effect. **F** PDIA4 promoter contains four latent binding sites with TDRKH-AS1. (Primers for the regions marked by purple). **G**, **H** ChIRP was performed followed by qPCR to verified that TDRKH-AS1 bound to the promoter of PDIA4. The specific binding of TDRKH-AS1 with its probes was verified using qRT-PCR. **I** Knockdown PDIA4 partially alleviated the pyroptosis-associated proteins expressions trigged by overexpression TDRKH-AS1 in HUVECs using western blotting. **J**–**L** The release of LDH, IL-1β and IL-18 induced by overexpression of TDRKH-AS1 were partially inhibited via knockdown of PDIA4 in HUVECs using LDH activity assay and ELISA. The data are represented as the mean ± SD; **p < 0.01, *p < 0.05

To further unveil the mechanism of TDRKH-AS1 in HUVECs, we performed RNA pull-down experiment following mass-spectrometry to detect proteins interacting with TDRKH-AS1 and found 138 proteins interact with TDRKH-AS1 (Fig. 6A, Additional file 1: Fig. S1D, Additional file 8: Table S7). These proteins were significant enriched in 3 pathways, including protein processing in endoplasmic reticulum, spliceosome and bacterial invasion of epithelial cells (Fig. 6B). The endoplasmic reticulum stress related gene protein disulfide isomerase family a member 4 (PDIA4) [36] was more abundant in the pulldown proteins detected by mass-spectrometry (Additional file 8: Table S7), suggesting that PDIA4 might play a major role in this regulatory network. Moreover, we confirmed the interaction between TDRKH-AS1 and PDIA4 using RNA pull-down followed by western blotting (Fig. 6C). RNA immunoprecipitation (RIP) and qRT-PCR were conducted to verify the interaction between TDRKH-AS1 and PDIA4 (Fig. 6D). The regulation of TDRKH-AS1 on PDIA4 was validated through qRT-PCR and western blotting, which showed increased mRNA and protein of PDIA4 (threefold and 1.56-fold) in HUVECs with TDRKH-AS1 overexpression, and decreased (89% and 47%) in HUVECs with TDRKH-AS1 knockdown (Fig. 6E). LncRNAs can bind promoters to regulate gene transcription [37]. Thus, we used LongTarget software[33] to predict TDRKH-AS1’s binding sites in the PDIA4 promoter, and found that TDRKH-AS1 had 4 potential binding sites in the PDIA4 promoter (Fig. 6F). We further confirmed the interaction between TDRKH-AS1 and the promoter of PDIA4 (region2: chr7:149028576-149028711) using chromatin Isolation by RNA Purification (ChIRP) followed by qPCR (Fig. 6G, H).

We further established overexpression and knockdown of PDIA4 in HUVECs for exploring the effect (Additional file 1: Fig. S1E). PDIA4 overexpression promoted twofold the expression levels of pyroptosis-associated proteins NLRP3, cleaved-caspase-1, ASC and GSDMD-NT (Fig. 6I). In addition, PDIA4 knockdown could partially mitigate ~ 16% of pyroptosis in HUVECs triggered by TDRKH-AS1 overexpression (Fig. 6I). Moreover, similar results were indicated that the LDH, IL-1β and IL-18 release induced by TDRKH-AS1 overexpression were alleviated by 19%, 62% and 33% after PDIA4 knockdown (Fig. 6J–L). These results suggest that TDRKH-AS1 may regulate the PDIA4 expression through binding to its promoter, leading to pyroptosis of HUVECs.

### TDRKH-AS1 trigged pyroptosis in HUVECs via recruiting PDIA4 to bind DDIT4

**Fig. 7:**
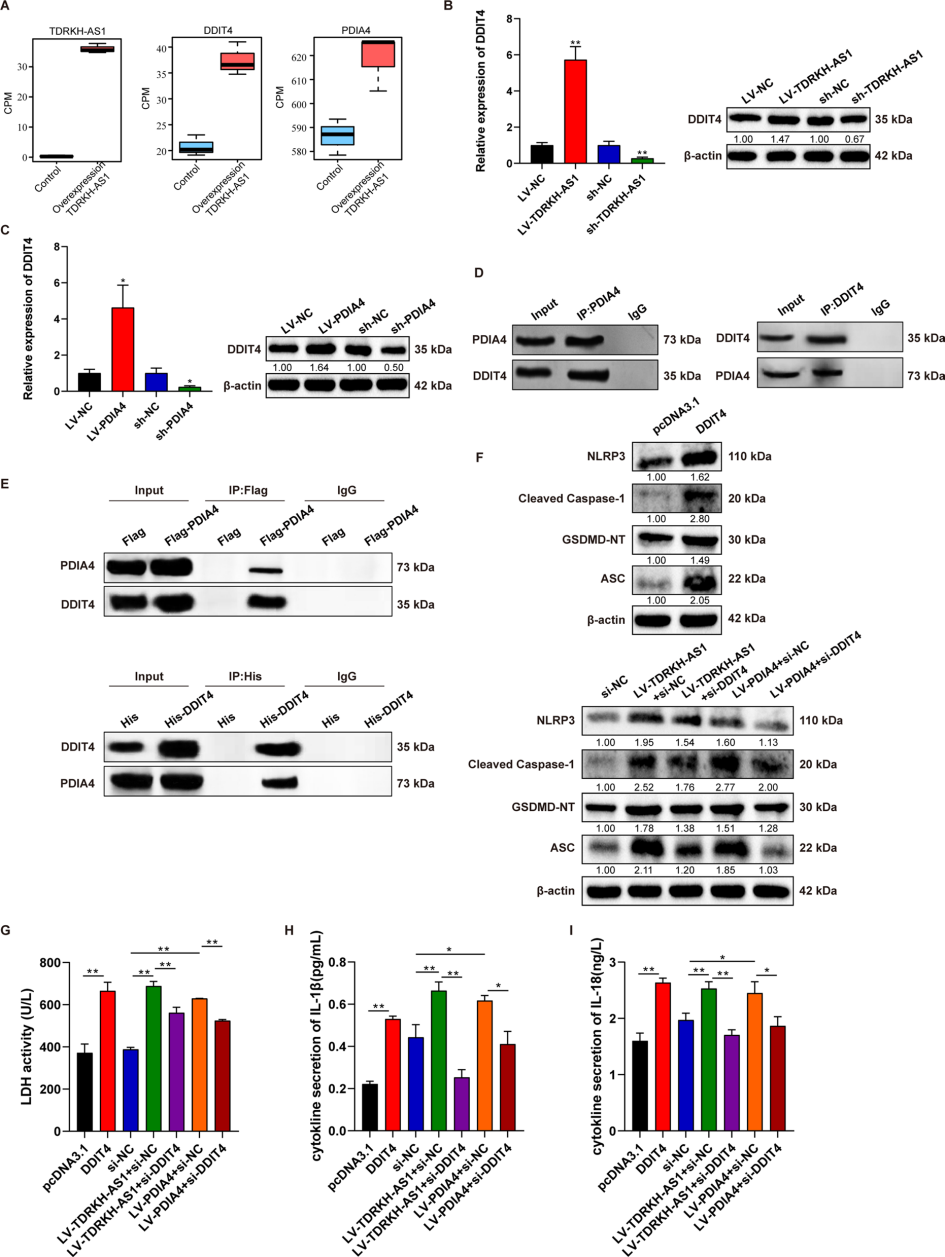
TDRKH-AS1 elevates the DDIT4 expression induced pyroptosis through PDIA4 binding. **A** TDRKH-AS1, DDIT4 and PDIA4 were upregulated in HUVECs with TDRKH-AS1 overexpression. **B**, **C** The expression of DDIT4 on mRNA and protein levels were increased by TDRKH-AS1 or PDIA4 overexpression, while the opposite results were obtained with TDRKH-AS1 or PDIA4 knockdown. **D**, **E** The interaction between PDIA4 and DDIT4 by Co-IP analyses and western blotting in HUVECs. **F** TDRKH-AS1 or PDIA4 overexpression increased the expression of pyroptosis-associated proteins and partially blocked with knockdown of DDIT4 using western blotting in HUVECs. **G**–**I** Knockdown of DDIT4 partially attenuated LDH, IL-1β and IL-18 release induced by overexpression TDRKH-AS1 or PDIA4 in HUVECs by LDH activity assay and ELISA. The data are represented as the mean ± SD; **p < 0.01, *p < 0.05

Based on the results RNA-seq on the HUVECs with TDRKH-AS1 overexpression, TDRKH-AS1, PDIA4 and DNA damage-inducible transcript 4 (DDIT4) were upregulated in HUVECs (Fig. 7A), consistent with its increased expression in HUVECs with TDRKH-AS1 overexpression (Fig. 6E). Among these DEGs, DDIT4 showed the most significant expression changes (1.8-fold increase) (Additional file 5: Table S4). Since DDIT4 are reported to be involved the activation of NLRP3 inflammasome and the inflammation-mediated endothelial cells injury process [38–41], upregulation of DDIT4 by TDRKH-AS1 might have similar functional effects on HUVECs. To further confirm the regulatory relationship among TDRKH-AS1, PDIA4 and DDIT4, we performed qRT-PCR and western blotting indicated that the mRNA and protein expression levels of DDIT4 were elevated by 3.6-fold and 3.1-fold in HUVECs with TDRKH-AS1 or PDIA4 overexpression. Opposite results were observed from studies on TDRKH-AS1 or PDIA4 knockdown cells with about 53% and 63% decrease (Fig. 7B, C). Moreover, co-immunoprecipitation (Co-IP) was also conducted to further investigate the potential interaction between PDIA4 and DDIT4. The interaction between endogenous PDIA4 and DDIT4 was confirmed by Co-IP assay and western blotting (Fig. 7D). Similar results were obtained in HUVECs transfected with PDIA4 or DDIT4 overexpression (Fig. 7E).

In order to further explore the mechanisms of pyroptosis induced by TDRKH-AS1 overexpression in HUVECs, the expression regulation of the downstream genes of DDIT4 were subsequently investigated. We established overexpression and/or knockdown DDIT4 in different combination in HUVECs (Additional file 1: Fig. S1F). DDIT4 overexpression elevated ~ twofold in the expression levels of pyroptosis-associated proteins and DDIT4 knockdown partially inhibited pyroptosis (about 30% decrease) that was initiated by TDRKH-AS1 or PDIA4 overexpression (Fig. 7F). Similarly, TDRKH-AS1 or PDIA4 overexpression promoted the release of LDH, IL-1β and IL-18 in HUVECs which could be alleviated (about 18%/17%, 62%/33%, and 33%/24%, respectively) by DDIT4 knockdown (Fig. 7G–I). These results indicated that TDRKH-AS1 could modulate the expression of DDIT4 through PDIA4 binding involved in pyroptosis of HUVECs.

**Fig. 8:**
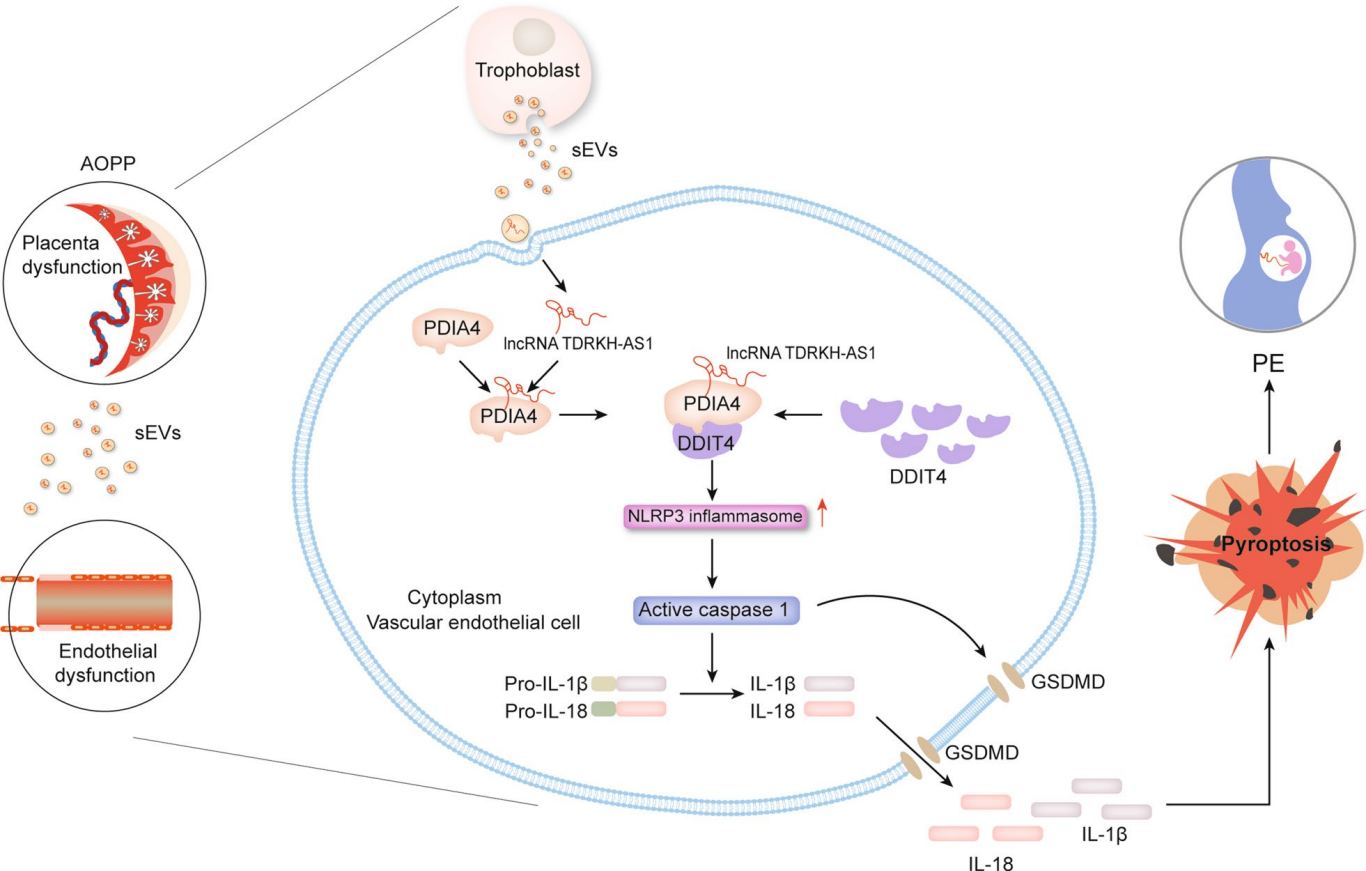
Schematic for illustrating potential role of sEVs-transported TDRKH-AS1 derived from AOPPs-treated trophoblasts in etiology of preeclampsia. sEVs-containing TDRKH-AS1 derived from AOPPs-treated trophoblasts may elevate the expression of DDIT4 through PDIA4 binding and trigger vascular endothelial cells pyroptosis in preeclampsia

Taken together, our findings demonstrate that sEVs-transported lncRNA TDRKH-AS1 derived from AOPPs-treated trophoblasts recruits the ER stress induced protein PDIA4 to bind DDIT4 and elevates the expression of this inflammation-related factor involved in vascular endothelial cells pyroptosis during preeclampsia (Fig. 8). A novel mechanism of sEVs-transported TDRKH-AS1 derived from AOPPs-treated trophoblasts to promote pyroptosis through PDIA4/DDIT4 axis may be crucial in the development of PE.

## Discussion

It is widely accepted that preeclampsia is not just a placental origin but particularly with suffering from trophoblast oxidative stress [3]. AOPPs, known as not only a critical product but also inducer of oxidative stress, is revealed to be involved in the progression of multiple chronic inflammatory disorders, especially PE [23, 25, 26]. Our previous studies have demonstrated that increased expression levels of AOPPs from plasma and placenta of PE patients show strongly association with the disease severity. Moreover, through rupture of oxidative pathway balance, AOPPs impair the function of trophoblast, leading to placenta damage [25, 26]. Placenta stress and endothelial dysfunction are inseparable causes, both of which play a potent role in the pathogenesis of PE. The factors from oxidative stress placenta are released in the maternal circulation, subsequently leading to excessive inflammation and systemic endothelial dysfunction [1, 2]. However, little is known about the components of these products generated by stress placenta and how they subvert the function of endothelial cells.

Abnormal expression of pEVs stimulated by oxidative stress placenta can transport complicated contents to maternal circulation, consequently resulting in endothelial injury and a potent inflammatory response of PE [11, 15]. Intriguingly, previous studies have shown that EVs-derived lncRNAs participate in the pathogenesis of PE via dysregulating the biological function of endothelial and trophoblast cells [16, 42]. It is conceivable that EVs-derived lncRNAs have the potential to be biomarkers in early diagnosis and to be the targets for therapy because of their good stability, as found in other various diseases [43]. Therefore, we have carried out lncRNA/mRNA sequencing on sEVs secreted from AOPPs-treated trophoblast and found 976 differentially expressed lncRNAs. TDRKH-AS1 as an antisense RNA is among the top upregulated lncRNAs. Its chromosomal location is 1q21.3 with three spliceosome. We checked the expression level of TDRKH-AS1 in plasmatic sEVs and placentae from EOSPE, LOSPE and controls, and found that it was significantly upregulated in PE patients. In addition, the expression level of TDRKH-AS1 in plasmatic sEVs was positively correlated with blood pressure of the patients. Our characteristic results confirmed the quality of sEVs and provided a solid foundation for the subsequent experiments. Encouragingly, substantial evidences have demonstrated that PE-like pregnant mice injected with pEVs from PE patients manifest hypertension, proteinuria, decreased body weight and changed vessels diameters in the placental labyrinth through inducing endothelial damage, vasoconstriction and inflammation activation [11–14]. Our in vivo results are consistent with the previous studies showing that the pregnant mice injected with TDRKH-AS1-riched sEVs, AOPPs-induced sEVs, AOPPs and L-NAME exhibited the above hallmark features of PE, structural labyrinth abnormalities and systemic inflammatory factors release such as IL-1β and IL-18. These findings suggest that oxidative stress trophoblast sEVs-derived TDRKH-AS1 may be involved in the pathogenesis of PE through triggering endothelial injury and inflammation activation.

To figure out what effects TDRKH-AS1 had in endothelial cells, the transcriptome sequencing of overexpression TDRKH-AS1 in HUVECs was conducted. We found 2030 DEGs, which were enriched in inflammatory signaling pathways, especially NOD-like receptor signaling pathway regulating pyroptosis. Strikingly, extensive research have suggested that pyroptosis, also termed inflammatory necrosis, plays a role in the pathogenesis of PE which trigger endothelial dysfunction and robust proinflammatory reactions [44]. As previously discovered, the expressions of pyroptosis-related mediators are markedly increased in placenta and peripheral blood from pregnant women with PE patients compared to healthy pregnant controls [28, 45], meanwhile the systemic manifestation of PE-like mice model is reversed by NLRP3 or capase-1 activity deficiency [12, 46]. Furthermore, pyroptosis is also considered to contribute importantly to endothelial cells death in various diseases [30]. In accordance with the above views, we demonstrated that overexpression TDRKH-AS1, TDRKH-AS1-riched sEVs, AOPPs-induced sEVs, and AOPPs could facilitate the expression levels of pyroptosis-related proteins such as NLRP3, cleaved-caspase-1, ASC and GSDMD-NT in HUVECs. Moreover, the plasma membrane integrity of HUVECs was beached with swelling and holes formation observed by SEM. As we know, pyroptosis is characterized by subsequent intracellular particulates release [29]. Our data showed that the release of LDH, IL-1β and IL-18 were elevated dramatically in HUVECs treated with overexpression TDRKH-AS1, TDRKH-AS1-riched sEVs, AOPPs-induced sEVs, and AOPPs. These results demonstrated that TDRKH-AS1 derived from trophoblast sEVs under oxidative stress triggered pyroptosis with Intensive inflammatory response and aggravated endothelial cells damage, which may consequently contribute in the development of PE, consistent with prior studies on endothelial injury induced by hypoxia-induced EVs [13, 16].

Mechanistically, 138 TDRKH-AS1-binding proteins we detected using RNA pull-down and mass spectrometry which significantly enriched in protein processing in endoplasmic reticulum, consistent with the sequencing of trophoblast sEVs induced by AOPPs. The endoplasmic reticulum maker PDIA4 as a stress-induced protein has been implicated in the pathogenesis of various diseases, such cardiovascular disorders and cancers [47, 48]. Moreover, IL-11 can induce endoplasmic reticulum response via regulating PDIA4 in PE [36]. Our results indicate that the lncRNA TDRKH-AS1 not only interact with protein PDIA4, but also binds to promoter of the gene *PDIA4*. The expression of PDIA4 was regulated by overexpression or knockdown TDRKH-AS1 in HUVECs. In addition, numerous studies have revealed that the endoplasmic reticulum is widely implicated in NLRP3 inflammasome activation, resulting in pyroptosis in multiple diseases including PE [28, 49, 50]. Endothelial inflammation and injury are triggered by NLRP3 inflammasome activation during endoplasmic reticulum stress [51]. We found that the pyroptosis-related proteins expressions, such as NLRP3, cleaved-caspase-1, ASC and GSDMD-NT were increased by PDAI4 overexpression. Conversely, PDAI4 knockdown could partially attenuate these proteins expressions by TDRKH-AS1 overexpression. Similar results were observed in LDH, IL-1β and IL-18 release detection. Therefore, it is reasonable to speculate that TDRKH-AS1 may induce endothelial cells pyroptosis by recruiting PDIA4 in PE.

DDIT4, a novel stress responsible marker, was the most significantly upregulated gene in the TDRKH-AS1 overexpression sequencing in HUVECs. Act as a molecular link between endoplasmic reticulum stress and inflammation, DDIT4 is engaged in the procession of various disease, such as cardiovascular disease and PE [38, 39, 52]. Additionally, elimination of DDIT4 can dramatically ameliorate the inflammatory response and endothelial injury, suggesting that DDIT4 may be a novel therapeutic target [38, 39]. Meanwhile, DDIT4 is a prominent modulator of NLRP3 inflammasome activation, subsequently leading to enormous inflammatory cytokines release [40, 41]. In our study, we found that the DDIT4 interacted directly with PDIA4, whose expression level was regulated by TDRKH-AS1 or PDIA4 overexpression and knockdown. Consistent with prior studies on the pyroptosis-related proteins, the expressions of NLRP3, cleaved-caspase-1, ASC and GSDMD-NT were apparently increased in HUVECs after DDIT4 overexpression. Furthermore, elimination of DDIT4 effectively inhibits the upregulation of pyroptosis-related proteins induced by TDRKH-AS1 or PDIA4 overexpression, concomitant with reduced levels of LDH, IL-1β and IL-18 release. Thus, it is tempting to speculate that the TDRKH-AS1-induced pyroptosis on endothelial cells, which may be initiated through the regulating the expression of DDIT4 via PDIA4 binding.

## Conclusions

Collectively, as a summary of our findings in current study, the expression levels of TDRKH-AS1 in sEVs isolated by AOPPs-induced trophoblast can be remarkably elevated, which have been identified as a potential causal factor for this disease. The aforementioned findings in vivo and vitro stimulated with TDRKH-AS1-rich sEVs have demonstrated that TDRKH-AS1 from AOPPs-treated trophoblast sEVs can trigger pyroptosis in endothelial cells with extensive inflammatory responses. TDRKH-AS1 recruits PDIA4 to upregulate DDIT4 expression, leading to pyroptosis of endothelial cells. Herein, we have demonstrated that trophoblast sEVs-derived TDRKH-AS1 initiates pyroptosis of endothelial cells and thus participates the pathogenesis of PE, providing new insights in developing early diagnosis and effective therapy of PE.

## Supplementary Information

**Additional file 1: Figure S1.** Detection of the expression levels of TDRKH-AS1 in cells and sEVs with different treatments. **A** The qRT-PCR quantification of TDRKH-AS1 expression in HTR8/SVneo cells transfected with full-length sequence of human TDRKH-AS1 and empty vector. **B** The qRT-PCR quantification of TDRKH-AS1 expression in sEVs and HUVECs treated with AOPPs and TDRKH-AS1 sEVs. **C** The qRT-PCR quantification of overexpress and knockdown TDRKH-AS1 expression in HUVECs transfected with lentivirus vectors. **D** The silvery staining of the gel with the proteins pulled down by TDRKH-AS1 and antisense RNA. **E** qRT-PCR and western blotting used to examine the PDIA4 expression levels transfected with overexpressing and knockdown lentivirus vectors in HUVECs. **F** The expression of DDIT4 detected by qRT-PCR and western blotting in HUVECs with transfection with pcDNA3.1- DDIT4 and siRNAs. **p < 0.01, *p < 0.05.

**Additional file 2: Table S1.** Clinical Characteristics of Normal Pregnancies and Preeclamptic.

**Additional file 3: Table S2.** Differentially expressed genes (DEGs) in sEVs derived from AOPPs-treated HTR8/SVneo cells. *p* value of both DESeq2 and edgeR less than 0.05 is considered to be differentially expressed genes.

**Additional file 4: Table S3.** Enriched KEGG pathways of DEGs in sEVs derived from AOPPs-treated HTR8/SVneo cells.

**Additional file 5: Table S4.** Differentially expressed genes (DEGs) induced by overexpression TDRKH-AS1 in HUVECs. *p* value of both DESeq2 and edgeR less than 0.05 is considered to be differentially expressed genes.

**Additional file 6: Table S5.** Enriched KEGG pathways of DEGs induced by overexpression TDRKH-AS1 in HUVECs.

**Additional file 7: Table S6.** Inflammatory and pyroptosis associated genes collected from Gene Ontology (GO), MSigDB and genecards databases.

**Additional file 8: Table S7.** Proteins interacting with TDRKH-AS1 detected by Mass Spectrometry and enriched KEGG pathways.

**Additional file 9: Table S8.** Sequences of primers and siRNA.

**Additional file 10: Table S9.** Sequences of TFO1, TTSs, probes and primers.

## Data Availability

The datasets used and/or analyzed during the current study are available from the corresponding author on reasonable request.

## Abbreviations

PE: Preeclampsia
AOPPs: Advanced oxidation protein products
EVs: Extracellular vesicles
MVs: Microvesicles
Exos: Exosomes
Abs: Apoptotic bodies
NLRP3: NOD-like receptor protein 3
GSDMD: Gasdermin D
LDH: Enzyme lactate dehydrogenase
TEM: Transmission electron microscopy
NTA: Nanoparticle tracking analysis
DEGs: Differentially expressed genes
SBP: Systolic blood pressure
HUVECs: Human umbilical vein endothelial cells
SEM: Scanning electronic microscopy
PDIA4: Protein disulfide isomerase family a member 4
RIP: RNA immunoprecipitation
DDIT4: DNA damage-inducible transcript 4
Co-IP: Co-immunoprecipitation
ELISA: Enzyme-linked immunosorbent assay
