# Supplementary material for: Small extracellular vesicles-transported lncRNA TDRKH-AS1 derived from AOPPs-treated trophoblasts initiates endothelial cells pyroptosis through PDIA4/DDIT4 axis in preeclampsia
Source: J Transl Med. 2023 Jul 24;21:496. doi: 10.1186/s12967-023-04346-6 (PMC10364420; doi:10.1186/s12967-023-04346-6)
Supplement: Supplementary file 1 — Additional file 1: Figure S1. Detection of the expression levels of TDRKH-AS1 in cells and sEVs with different treatments. A The qRT-PCR quantification of TDRKH-AS1 expression in HTR8/SVneo cells transfected with full-length sequence of human TDRKH-AS1 and empty vector. B The qRT-PCR quantification of TDRKH-AS1 expression in sEVs and HUVECs treated with AOPPs and TDRKH-AS1 sEVs. C The qRT-PCR quantification of overexpress and knockdown TDRKH-AS1 expression in HUVECs transfected with lentivirus vectors. D The silvery staining of the gel with the proteins pulled down by TDRKH-AS1 and antisense RNA. E qRT-PCR and western blotting used to examine the PDIA4 expression levels transfected with overexpressing and knockdown lentivirus vectors in HUVECs. F The expression of DDIT4 detected by qRT-PCR and western blotting in HUVECs with transfection with pcDNA3.1- DDIT4 and siRNAs. **p < 0.01, *p < 0.05. [file 12967_2023_4346_MOESM1_ESM.pdf]

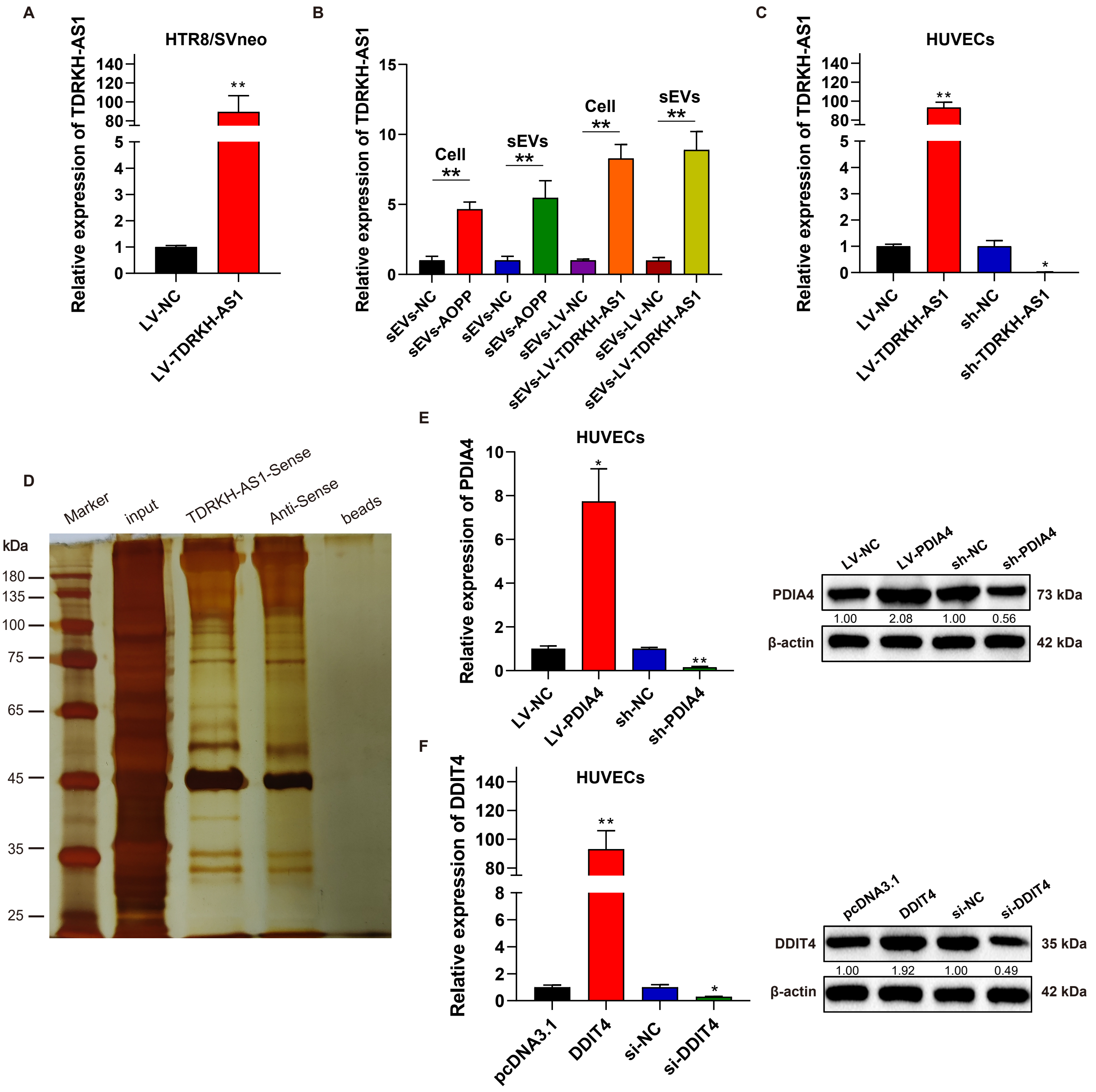
A
HTR8/SVneo
Relative expression of TDRKH-AS1
140
120
100
80
5
4
3
2
1
0
**
LV-NC
LV-TDRKH-AS1
B
Relative expression of TDRKH-AS1
15
10
5
0
Cell
**
sEVs
**
Cell
**
sEVs
**
sEVs-NC
sEVs-AOPP
sEVs-NC
sEVs-AOPP
sEVs-LV-NC
sEVs-LV-TDRKH-AS1
sEVs-LV-NC
sEVs-LV-TDRKH-AS1
C
HUVECs
Relative expression of TDRKH-AS1
140
120
100
80
5
4
3
2
1
0
**
*
LV-NC
LV-TDRKH-AS1
sh-NC
sh-TDRKH-AS1
D
Marker
input
TDRKH-AS1-Sense
Anti-Sense
beads
kDa
180
135
100
75
65
45
35
25
E
HUVECs
Relative expression of PDIA4
10
8
6
4
2
0
*
**
LV-NC
LV-PDIA4
sh-NC
sh-PDIA4
LV-NC
LV-PDIA4
sh-NC
sh-PDIA4
PDIA4
73 kDa
1.00
2.08
1.00
0.56
β-actin
42 kDa
F
HUVECs
Relative expression of DDIT4
140
120
100
80
8
6
4
2
0
**
*
pcDNA3.1
DDIT4
si-NC
si-DDIT4
pcDNA3.1
DDIT4
si-NC
si-DDIT4
DDIT4
35 kDa
1.00
1.92
1.00
0.49
β-actin
42 kDa

## Supplemental Figure Legends

**Figure S1. Detection of the expression levels of TDRKH-AS1 in cells and sEVs with different treatments.**

(A) The qRT-PCR quantification of TDRKH-AS1 expression in HTR8/SVneo cells transfected with full-length sequence of human TDRKH-AS1 and empty vector.

(B) The qRT-PCR quantification of TDRKH-AS1 expression in sEVs and HUVECs treated with AOPPs and TDRKH-AS1 sEVs.

(C) The qRT-PCR quantification of overexpress and knockdown TDRKH-AS1 expression in HUVECs transfected with lentivirus vectors.

(D) The silvery staining of the gel with the proteins pulled down by TDRKH-AS1 and antisense RNA.

(E) qRT-PCR and western blotting used to examine the PDIA4 expression levels transfected with overexpressing and knockdown lentivirus vectors in HUVECs.

(F) The expression of DDIT4 detected by qRT-PCR and western blotting in HUVECs with transfection with pcDNA3.1- DDIT4 and siRNAs.

**$p < 0.01$, *$p < 0.05$.

## Supplemental Table Legends

**Table S1: Clinical Characteristics of Normal Pregnancies and Preeclamptic.**

**Table S2: Differentially expressed genes (DEGs) in sEVs derived from AOPPs-treated HTR8/SVneo cells. *p* value of both DESeq2 and edgeR less than**

**0.05 is considered to be differentially expressed genes.**

**Table S3: Enriched KEGG pathways of DEGs in sEVs derived from AOPPs-treated HTR8/SVneo cells.**

**Table S4: Differentially expressed genes (DEGs) induced by overexpression TDRKH-AS1 in HUVECs. *p* value of both DESeq2 and edgeR less than 0.05 is considered to be differentially expressed genes.**

**Table S5: Enriched KEGG pathways of DEGs induced by overexpression TDRKH-AS1 in HUVECs.**

**Table S6: Inflammatory and pyroptosis associated genes collected from Gene Ontology (GO), MSigDB and genecards databases.**

**Table S7: Proteins interacting with TDRKH-AS1 detected by Mass Spectrometry and enriched KEGG pathways.**

**Table S8: Sequences of primers and siRNA.**

**Table S9: Sequences of TFO1, TTSs, probes and primers.**
