# Supplementary material for: Small extracellular vesicles-transported lncRNA TDRKH-AS1 derived from AOPPs-treated trophoblasts initiates endothelial cells pyroptosis through PDIA4/DDIT4 axis in preeclampsia
Source: J Transl Med. 2023 Jul 24;21:496. doi: 10.1186/s12967-023-04346-6 (PMC10364420; doi:10.1186/s12967-023-04346-6)
Supplement: Supplementary file 9 — Additional file 9: Table S8. Sequences of primers and siRNA. [file 12967_2023_4346_MOESM9_ESM.docx]

**Table S8**： Sequences of primers and siRNA.

| Primers | Sense(5’-3’) | Antisense(5’-3’) |
| --- | --- | --- |
| ACTB | CTCCATCCTGGCCTCGCTGT | GCTGTCACCTTCACCGTTCC |
| PDIA4 | GGCAGGCTGTAGACTACGAG | TTGGTCAACACAAGCGTGACT |
| DDIT4 | TGAGGATGAACACTTGTGTGC | CCAACTGGCTAGGCATCAGC |
| TDR-AS1-1 | ATAGGCTTTGTAGATGGACAGGAAGT | CTCCTGCCGCTGTTTCG |
| TDR-AS1-2 | TGCACAAATGACAAGTATATATCTTGATG | GAGAAGAGCAGTGACTGAGAGGG |
| U1 | GGGAGATACCATGATCACGAAGGT | CCACAAATTATGCAGTCGAGTTTCCC |
| siRNA | Sense(5’-3’) | Antisense(5’-3’) |
| DDIT4 | CUGCUGAUGCCUAGCCAGUUGGUAATT | UUACCAACUGGCUAGGCAUCAGCAGTT |
