# Supplementary material for: Small extracellular vesicles-transported lncRNA TDRKH-AS1 derived from AOPPs-treated trophoblasts initiates endothelial cells pyroptosis through PDIA4/DDIT4 axis in preeclampsia
Source: J Transl Med. 2023 Jul 24;21:496. doi: 10.1186/s12967-023-04346-6 (PMC10364420; doi:10.1186/s12967-023-04346-6)
Supplement: Supplementary file 10 — Additional file 10: Table S9. Sequences of TFO1, TTSs, probes and primers. [file 12967_2023_4346_MOESM10_ESM.docx]

**Table S9**： Sequences of TFO1, TTSs, probes and primers.

| TFO1 | Sequences | |
| --- | --- | --- |
| TFO1 | TTTTTTAAATTGTATACATTATGGTTTTTTTTTTT | |
| TTSs | Sequences | |
| TTS1 | TCGGCACCCGCCACGGCCAGCAGCTGCACCAGCCCCAAGAGCAGCAG | |
| TTS2 | TTTCCGGGGCCTCATGGTAGCGGGGGCGGAGCGCGGCCTCCTAGCGTCGGCGGCCGCTGAGCGCACCGA | |
| TTS3 | ACGCCGGCACGGACCGCGCGCGCCTCGGCCGCGACG | |
| TTS4 | GCTCCGCCGCCCTAGGCCCTCCCTACTCCTGCGCG | |
| Probes  (TDRKH-AS1) | Sequences | |
| 1 | CATGATTCAACGCTCTCATG | |
| 2 | CTGATGGAGCATCGCCCATC | |
| 3 | GCTTGTCCCTTGACTTCCTG | |
| 4 | TGCACTCCAGCCTGGGTGAC | |
| 5 | GGTGTGGTGGTGGGCACCTG | |
| 6 | CGCAGTGGCTCATGCCTGTA | |
| 7 | ACAAATGTATTCCATTTGTG | |
| 8 | GGTATCAGGAGTCAGAAGAG | |
| Primers | Sense(5’-3’) | Antisense(5’-3’) |
| TDRKH-AS1 | ATAGGCTTTGTAGATGGACAGGAAGT | CTCCTGCCGCTGTTTCG |
| Region 1 | GCTTGCCGCTCACCCTC | CGTCGCTAAGGAGCGACG |
| Region 2 | CCAATCCCAGACTGACGC | CACCTCACGTTAGGGCTCG |
| GAPDH | GTCGGAGTCAACGGATTTG | TGGGTGGAATCATATTGGAA |
